# Complicated Native Aortic Valve Endocarditis: Complexities of Medical Optimisation Prior to Surgical Repair for Large Vegetations With Systemic Emboli

**DOI:** 10.7759/cureus.42718

**Published:** 2023-07-31

**Authors:** Lucas Jullian, Josh Davies, Mansoor Zafar, Mithilaa Senthivel, Jad Alkhoury

**Affiliations:** 1 Cardiology, Conquest Hospital, East Sussex Healthcare National Health Service (NHS) Trust, St. Leonards-on-Sea, GBR; 2 Internal Medicine, Conquest Hospital, East Sussex Healthcare National Health Service (NHS) Trust, St. Leonards-on-Sea, GBR; 3 Gastroenterology, General Internal Medicine, Conquest Hospital, East Sussex Healthcare National Health Service (NHS) Trust, St. Leonards-on-Sea, GBR

**Keywords:** medical optimisation, infective endocarditis, staphylococcus aureus, acute ischaemic infarcts, non-coronary cusp of aortic valve

## Abstract

A 43-year-old male with no history of valvular disease but ongoing intravenous drug use presented with acute confusion, pyrexia, and Osler’s nodes. Transthoracic echocardiography uncovered a large 17 x 15 mm-sized vegetation on the aortic valve, causing moderate-to-severe aortic regurgitation. Subsequent multi-organ compromise and complexities regarding treatment adherence delayed surgical intervention; thus, six weeks of antibiotic therapy and medical optimisation, in close collaboration with cardiology, microbiology, and cardiothoracic teams, enabled definitive aortic valve repair to be performed. This case highlights the challenges encountered when managing this life-threatening condition and the obstacles of enacting the guidelines recommendations regarding the timing of surgical intervention. Our case portrays the effectiveness of medical management as bridge-to-surgery in patients not in a position to undergo immediate surgical repair.

## Introduction

Despite the rise of detailed expert guidelines on the presentation and management of infective endocarditis (IE), the in-hospital mortality of patients presenting with this condition remains 20-30%, largely unchanged since the assertion of surgical treatment in the 1960s [1]. This is attributable to the severe complications arising from this disease, ranging from cerebrovascular embolic events, progressive heart failure, and irreversible structural damage resulting from uncontrolled valvular infection [2]. To prevent and address this life-threatening condition, the European Society of Cardiology developed guidance to encourage urgent cardiac surgery, defined as surgery within seven days of presentation, for aortic native-valve or prosthetic valve endocarditis with evidence of heart failure, locally uncontrolled infection, or with large vegetations [3]. However, there is a significant proportion of patients meeting those criteria whose surgical intervention is delayed or declined, as a consequence of haemodynamic instability, poor prognosis, or uncertainties regarding adherence to treatment [4]. We present a case of acute IE in a patient with multiple embolic and immunological complications, as well as large and mobile aortic valve vegetations on echocardiography, complicated by multi-organ failure. This case sheds light on the complexities of managing similar patients with ongoing intravenous drug use, where medical management remains key but extremely challenging as a bridge-to-surgical intervention, and highlights the significance of effective multi-disciplinary involvement during the acute phase of complicated native-valve endocarditis.

## Case presentation

A 43-year-old man with a background of ongoing intravenous drug use presented to the emergency department with a one-week history of confusion, worsening agitation, malaise, and a venous ulcer to his left shin. Examination found a harsh ejection-systolic murmur with a soft diastolic murmur, splinter haemorrhages in all extremities, and tender popular lesions on both hands' index finger and thumb, in keeping with Osler’s nodes. Although haemodynamically stable, he presented a Glasgow Coma Scale of 14, with low-grade pyrexia, and was noted to be pale and sweaty on admission.

His past medical history consists of previous deep vein thrombosis and a diagnosis of hepatitis C with associated liver cirrhosis 10 years prior to this admission, with repeated failure to attend appointments and adhere to treatment with a high re-infection risk due to concurrent drug use.

His blood tests on admission portrayed neutrophilia with mild normocytic anaemia, accompanied by a raised D-dimer and acute kidney injury stage 1 (Table 1).

**Table 1:** Blood test results on admission CRP: C-reactive protein, eGFR: estimated glomerular filtration rate, INR: international normalised ratio Source: Laboratory at Conquest Hospital, East Sussex Healthcare National Health Service (NHS) Trust *Procalcitonin interpretation guide Procalcitonin <0.10 ug/L - antibiotics strongly discouraged Procalcitonin 0.10-<0.25 ug/L - antibiotics discouraged Procalcitonin 0.25-0.50 ug/L - antibiotics encouraged Procalcitonin >0.50 ug/L - antibiotics strongly encouraged

Lab parameter	Units of measurement	Normal reference range	Patient’s blood test results
Serum sodium	mmol/L	133-146	141
Serum potassium	mmol/L	3.5-5.3	3.4
Serum urea	mmol/L	2.5-7.8	9
Serum creatinine	umol/L	59-104	202
CRP	mg/L	0-5	107
eGFR	mL/min/1.73m2	>70	34
Procalcitonin	ug/L	<0.10^*^	0.34
Haemoglobin	g/L	130-180	89
Mean cell volume	fl	80-100	91.9
White cell count	10^9^/L	4-11	12.71
Neutrophils	53	2-7.5	9.28
INR	-	0.8-1.2	1.3
D-dimer	ng/mL	0-225	801

**Figure 1:**
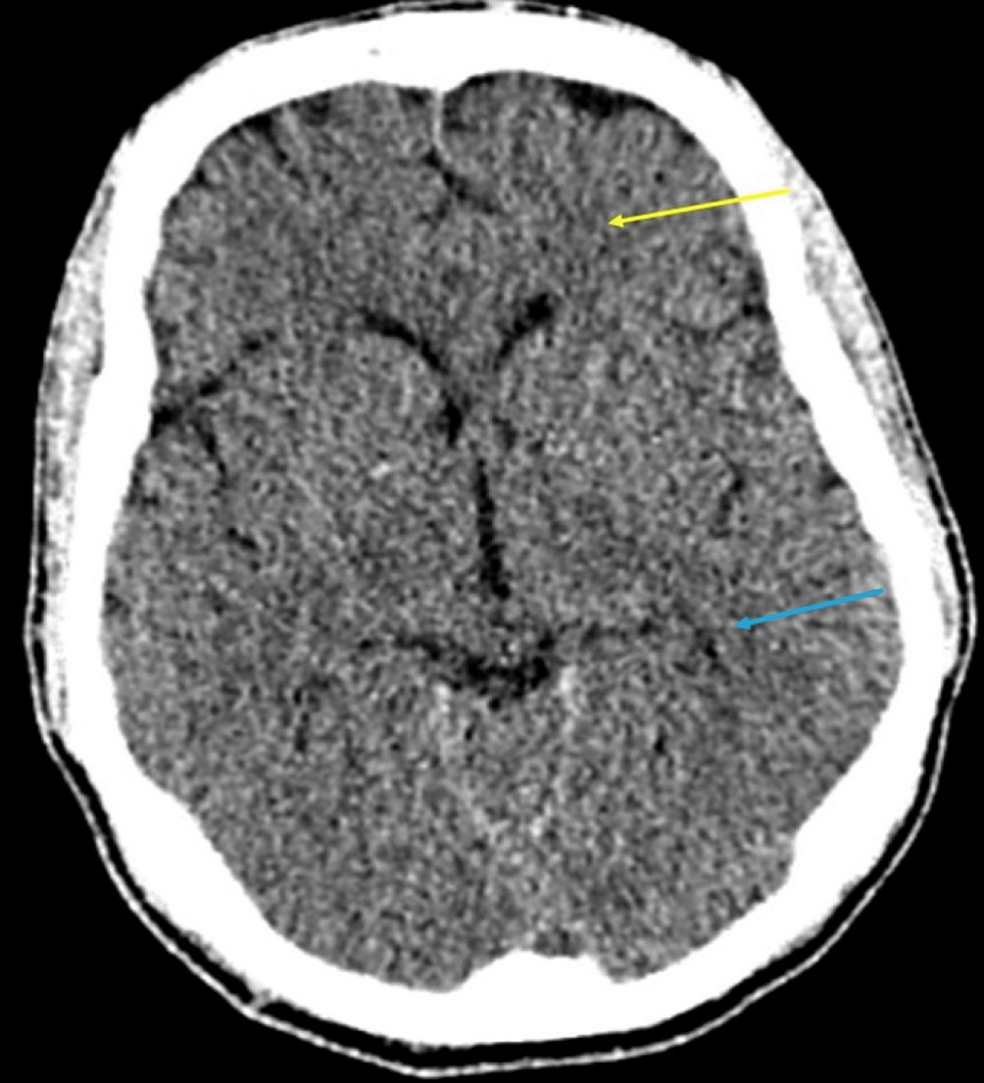
CT head with ill-defined hypodensities in the left frontal (yellow arrow) and parietal lobes (blue arrow) concerning acute ischaemic infarcts

Peripheral blood culture grew *Staphylococcus aureus* sensitive to flucloxacillin, and non-contrast CT head showed multiple left cerebral frontal and parietal lobe ischaemic infarcts (Figure 1).

Admission ECG showed sinus rhythm with no PR-interval prolongation. Differential diagnoses on admission were infective endocarditis with embolic and immunological complications, cerebral emboli due to ongoing intravenous drug use, and septicaemia secondary to an untreated left leg ulcer. Subsequently, serial blood cultures were obtained, an intravenous flucloxacillin regime was commenced, and early liaison with the cardiology and microbiology team was established, who advised optimising antibiotics treatment with dual therapy consisting of intravenous flucloxacillin and linezolid.

**Figure 2:**
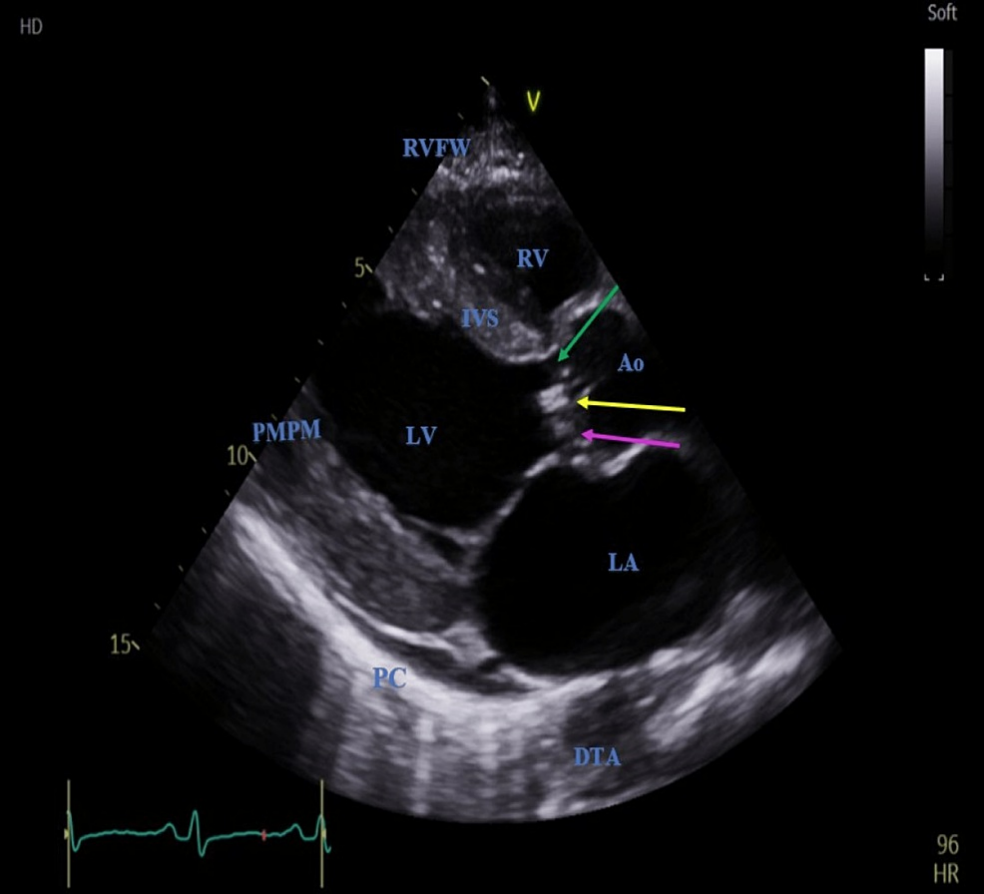
Parasternal long-axis view showing mobile, echogenic attachment on the aortic valve (yellow arrow) in between the right coronary cusp (green arrow) and non-coronary cusp (pink arrow) RV: right ventricle, RVFW: right ventricular free wall, IVS: interventricular septum, LV: left ventricle, PMPM: posteromedial papillary muscle, Ao: ascending aorta, LA: left atrium, PC: pericardium, DTA: descending thoracic aorta

The formal echocardiogram highlighted a large (17 x 15 mm) mobile and oscillating echogenic structure attached to the non-coronary cusp of the aortic valve (Figure 2, Video 1).

**Video 1:** Large mobile and oscillating echogenic structure attached to the non-coronary cusp of the aortic valve

**Figure 3:**
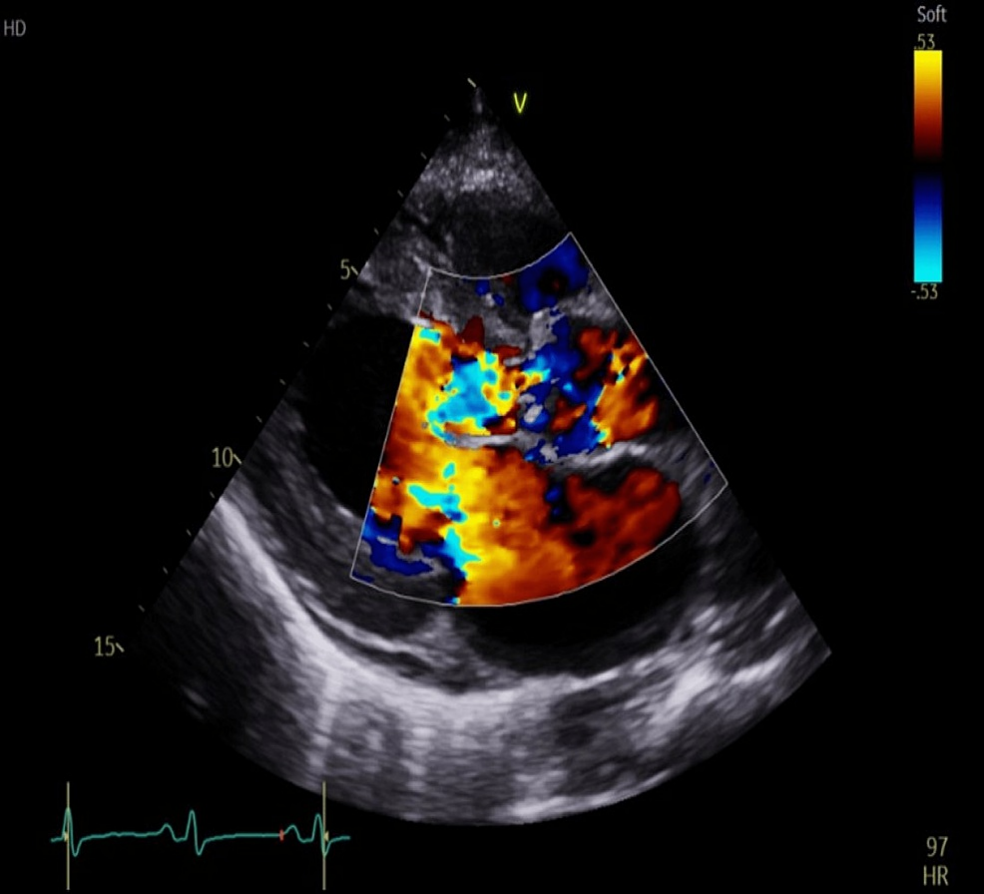
Parasternal long axis ECHO with colour flow showing severe aortic regurgitation

The systolic function of the left ventricle was, however, preserved (left ventricular ejection fraction 55-60%) with no other valvular abnormalities or right ventricular dysfunction except for moderate-to-severe aortic regurgitation and eccentric left ventricular hypertrophy on colour flow ECHO (Figure 3).

All three peripheral blood cultures were positive for flucloxacillin-sensitive *Staphylococcus aureus*. Two days following admission and intravenous antibiotics, the patient’s renal function significantly worsened with markedly deranged liver function tests, signifying his rapid deterioration as a result of the large vegetation with embolic events complicated by severe aortic regurgitation. Discussions with cardiothoracic surgeons yielded that due to the patient’s liver cirrhosis without previous oesophageal-gastro-duodenoscopy (OGD) and mortality risk due to multi-organ failure, initial conservative management was most appropriate until OGD was carried out to rule out any oesophageal varices and haemodynamic stability was achieved. A third antibiotic agent, rifampicin, was added to optimise medical management as a bridge to surgery.

Over the course of the next 10 days, the patient self-discharged against medical advice twice, the latter of which he re-presented to the hospital with significant shortness of breath and bilateral leg pitting oedema up to the mid-thighs and reported melaena. He was haemodynamically unstable, maintaining oxygen saturation of 94% on a 15 litre/minute non-rebreather mask, heart rate of 118, and blood pressure of 98/59. Chest X-ray suggested pulmonary oedema with possible right-sided consolidation. His haemoglobin significantly dropped to 33 with mixed respiratory and metabolic acidosis on arterial blood gas. Consequently, resuscitation using blood transfusion and cautious intravenous furosemide infusion was commenced with gradual improvement in clinical status over three weeks and no further haemorrhage. He underwent OGD a few days later which ruled out oesophageal varices but showed a solitary duodenal ulcer treated with a course of intravenous proton-pump inhibitor.

On completion of the six-week regime of triple antibiotic therapy, the patient’s renal function normalised with haemodynamic stability and repeat blood cultures were negative for microorganisms. A repeat echocardiogram showed no improvement in the size of the vegetation or function of the aortic valve, and, therefore, the cardiothoracic team concluded that the chance of survival without aortic valve replacement was minimal, and the patient was subsequently transferred to the local tertiary centre for aortic valve replacement following successful medical management as bridge-to-surgery.

## Discussion

Infective endocarditis (IE) refers to an infection arising from the innermost layer of the heart endothelium, including the heart valves. Circulating bacteria, commonly introduced via the bloodstream, adhere to the valvular endothelial cells and triggers a cascade of clotting and immunological phenomena. This results in the propagation of fragile vegetations which can sever and embolise, lodging its fragments through the distal circulation, causing a number of embolic and immunological complications commonly seen in IE [5]. With the increasing contact with healthcare-related facilities, along with the rise of intravenous drug use within the general population, the most common culprit of native-valve endocarditis in the Western World is now *Staphylococcus aureus* [1,6] affecting over 30% of all cases with native-valve endocarditis. This can be explained by the increased use of intravenous therapy during hospitalisation, the use of intravenous chemotherapy agents, dialysis, and long-term residence in care facilities [6]. This epidemiological finding is important as IE caused by *Staphylococcus aureus* is linked to higher mortality rates and the need for surgical intervention [7].

The most susceptible heart valve for IE depends on the mechanism of infection, irrespective of any pre-existing valvular disease; although tricuspid valve IE represents over half of the confirmed cases amongst patients affected by drug use, 20% of patients within this sub-group suffer from aortic valve IE [8]. Defining the infected valve is invaluable, as it allows us to predict and promptly diagnose the subsequent embolic complications seen in such cases.

Due to its insidious progression and growth of infective vegetations, often mobile and prone to embolisation, the clinical presentation of IE remains challenging [9]. It may present as an acute and progressive condition, with over 20% of cases displaying signs of embolic complications on presentation [10] but also as a subacute or chronic disease characterised by persistent fever with systemic stigmata of chronic illness, such as weight loss and fatigue [11]. As peripheral stigmata of IE including dermatologic presentations have become increasingly rare [12] with the reduced time delay between the onset of disease and presentation to the hospital, it appears primordial to consider infective endocarditis as a differential diagnosis in all patients presenting with fever and signs of embolic complications, to allow prompt initiation of antibiotic therapy and likely definitive surgical treatment in order to avoid life-threatening and often irreversible structural damage to the heart. Table 2 outlines the common embolic complications to major organs encountered in IE [11,13-14].

**Table 2:** The common embolic complications of IE and its clinical features IE: infective endocarditis *Left-sided IE refers to emboli originating from the mitral or aortic valve; right-sided IE refers to emboli originating from the tricuspid or pulmonary valve [11,13-14]

Site of embolic complication in IE	Possible complication	Common clinical features of embolic complication	Origin of embolus
Brain	Ischaemic stroke, cerebral abscess	New focal neurological sign, higher cognitive dysfunction	Left-sided IE*
Spleen	Splenic infarct, splenic abscess	Silent (30% of patients), left-upper quadrant abdominal pain	Left-sided IE
Kidneys	Renal infarct, acute renal failure	Decreased urine output, peripheral fluid retention	Left-sided IE
Lungs	Pulmonary embolism	Shortness of breath, pleuritic chest pain	Right-sided IE*

The risk of embolisation depends on several factors, including particular microorganisms (including *Staphylococcus aureus*), vegetations >10 mm in length, and multivalvular endocarditis [11]. Due to the aforementioned detection challenge of this condition, diagnosis continues to rely upon the modified Duke’s Criteria since the early 2000s [12], with echocardiography and positive blood cultures at the forefront of diagnosis. However, the sensitivity of trans-thoracic echocardiography (TTE) for vegetations and abscesses in native valves is only 70% and 50%, respectively, and, therefore, a negative TTE in the presence of clinical or microbiological suspicion of IE should not rule out endocarditis, where trans-oesophageal echocardiography should be considered where sensitivity for vegetation and abscesses for native valves is much higher at 96% and 90%, respectively [15].

Significant emphasis is placed upon prompt diagnosis and initiation of treatment, through antibiotic therapy and surgical intervention, as well as early discussion between cardiologists, microbiologists, and cardiac surgeons. This approach is justified by the exceptionally likely complications associated with aortic valve IE, often life-threatening and irreversible. Heart failure, resulting from worsening aortic valve regurgitation due to uncontrolled infection, represents the most common and most important variable predicting the in-hospital and one-year mortality of IE [2]. Embolic complications can also arise after the initiation of antibiotic therapy, as well as intracranial infectious aneurysms and acute renal failure. In such scenarios, renal failure will often be multi-factorial, resulting from septic emboli, haemodynamic impairment in those with heart failure or sepsis, antibiotic nephrotoxicity, and toxicity of contrast agents used during imaging procedures [16]. Not only do each of those increase the in-hospital mortality and morbidity, but developing any of these complications increases the peri-operative risk of surgical treatment, potentially jeopardising the only definite treatment option in the large majority of patients. Consequently, the European Society of Cardiology has developed clear recommendations regarding the timing and indications of surgery for patients presenting with left-sided endocarditis (Table 3) [9].

**Table 3:** Table collated, displaying a summarised and adapted version of the indications and timing of surgery in left-sided endocarditis. Selected indications are those labelled Class I, meaning ‘recommended’ based on large non-randomised studies, as per the European Society of Cardiology [9]

Indications for surgery	Timing of surgery
Aortic or mitral valve endocarditis with signs of pulmonary oedema or cardiogenic shock	Within 24 hours of diagnosis
Aortic or mitral valve endocarditis with symptoms of heart failure or poor haemodynamic tolerance	Within 7 days of diagnosis
Locally uncontrolled infection (abscess, enlarging vegetation of fistula)	Within 7 days of diagnosis
Aortic or mitral valve endocarditis with persistent vegetations >10 mm after one or more embolic episodes	Within 7 days of diagnosis

Surgical intervention is required for curative therapy in up to 50% of patients hospitalised with IE [17]. Co-morbidity is an important factor, such as concomitant liver or renal impairment, which may reduce the post-operative prognosis for surgical intervention and increase the risk of surgical complications due to haemodynamic compromise [18]. Several considerations may complicate this decision, as 10-year surgical mortality can exceed 60% [19]. Lastly, in the past, patients with a history of intravenous drug use were found to have infective endocarditis affecting the tricuspid valve from staphylococcal infection with a relatively better outcome [20]. However, the current trend is the involvement of the left-sided valves also, with or without a history of intravenous drug usage, with rather relatively lesser favourable outcomes. The high burden of comorbidities and antibiotic resistance are contemplated as possible reasons [21].

Patients affected by intravenous drug use also present with peculiar difficulties, such as uncertainty regarding adherence to treatment and recurrence of infection due to continued drug use [19]. In these cases, medical therapy with antibiotic treatment and regular blood culture monitoring may be preferred, pending a definitive decision between the patient, cardiologists, microbiologists, and cardiac surgeons. Should these considerations be deemed to exceed the therapeutic effect of surgical treatment, such patients may be treated with conservative medical management. These decisions are incredibly complex to manoeuvre, as highlighted in our case, and require early and regular discussion as a multi-disciplinary team, to assess all aspects of potential recovery benefits and risks involved, including the risk of re-infection and adherence to treatment.

## Conclusions

This clinical case offers an opportunity to highlight several considerations regarding the assessment and management of IE. Firstly, patients with a history of IE can have involvement of left-sided heart valves. Secondly, the initial presentation of patients later diagnosed with IE can vary immensely as a result of embolic complications suffered, and, therefore, we encourage clinicians to keep this condition as a differential in a wide range of clinical scenarios. Thirdly, although surgical intervention is advised within seven days of diagnosis in patients with large vegetation or symptoms of cardiac failure, this may be difficult to achieve due to the patient’s co-morbidities and haemodynamic status. Finally, our case reveals the value of coordinated medical management to enable such patients to undergo definite surgical intervention outside of the recommended window if necessary.
